# I’m going to fail! Acute cognitive performance anxiety increases threat-interference and impairs WM performance

**DOI:** 10.1371/journal.pone.0210824

**Published:** 2019-02-07

**Authors:** Angelos Angelidis, Ericka Solis, Franziska Lautenbach, Willem van der Does, Peter Putman

**Affiliations:** 1 Institute of Psychology, Leiden University, Leiden, The Netherlands; 2 Leiden Institute for Brain and Cognition, Leiden, The Netherlands; 3 Department of Psychiatry, Leiden University Medical Center, Leiden, The Netherlands; 4 Institute of Psychology, German Sport University Cologne, Cologne, Germany; 5 Institute of Sport Psychology and Sport Pedagogy, Leipzig University, Leipzig, Germany; Technion Israel Institute of Technology, ISRAEL

## Abstract

Stress can impair cognitive performance, as commonly observed in cognitive performance anxiety (CPA; e.g., test anxiety). Cognitive theories indicate that stress impairs performance by increasing attention to negative thoughts, a phenomenon also known as threat-interference. These theories are mainly supported by findings related to self-report measures of threat-interference or trait anxiety. Our main aim was to test, for the first time in a single study, the hypotheses that acute CPA-related stress negatively affects both working memory (WM) performance and objectively assessed threat-interference during performance. In addition, we aimed to assess the validity of a new stress-induction procedure that was developed to induce acute CPA. Eighty-six females were randomly assigned to a CPA-related stress group (*n* = 45) or a control group. WM performance and threat-interference were assessed with an *n*-back task (2-back and 3-back memory loads), using CPA-related words as distracters. The stress group showed higher state anxiety and slower WM performance. Both effects were moderated by trait CPA: the effects were stronger for individuals with higher trait CPA. Finally, trait CPA moderated the effect of stress on threat-interference during higher cognitive load: individuals with higher trait CPA in the stress group showed higher threat-interference. We conclude that acute CPA increases threat-interference and impairs WM performance, especially in vulnerable individuals. The role of threat-interference, cognitive load, and trait anxiety should be taken into account in future research. Finally, our method (combining our stressor and modified *n*-back task) is effective for studying stress-cognition interactions in CPA.

## Introduction

Almost every person will face many evaluative situations in life where optimal performance is required and where the result may be consequential, such as exams. Between 10 and 40 percent of students suffer to some degree from test anxiety [1,2]. An even higher prevalence has been found in subgroups such as students with disabilities, women, or minority students [3,4,5,6]. Many students fail in crucial exams due to performance anxiety, despite their ability to do well [7]. Test anxiety is part of the broader phenomenon of performance anxiety, which includes public speaking, stage fright and writing block. Performance anxiety refers to the anxiety that people experience in anticipation of and/or during important tasks, resulting in impaired performance [7]. In this study, the term cognitive performance anxiety (CPA) will be used to refer to the anxiety that arises during demanding cognitive tasks while people are or think that they are under evaluation, such as in test anxiety (cf., [8]). This may occur in any field, including academia (e.g., test anxiety) or work (e.g., public speaking anxiety or an office clerk worrying about a negative evaluation). Moderate levels of stress, including CPA, enhance cognitive performance (e.g., [9.]), but higher levels have detrimental effects [10,11].

There is increased interest in the mechanisms underlying the effects of stress on executive cognitive performance. Anxious people automatically scan their environment for threat cues, which then capture their attention, making it more difficult to disengage from this information (e.g., [12,13]). Thoughts about threat may also be processed automatically and preferentially [14,15]. Such cognitive interference by threat-related information has been given increased attention as it is suggested to play a crucial role in the development and/or maintenance of anxiety disorders (e.g., [16,17,18]; for a recent review, see [13]). The cognitive interference theory [19] states that anxiety impairs cognitive performance by increasing cognitive interference. The attentional control theory (ACT; [14]) further explains the mechanism of this relationship by introducing the role of attentional control. Specifically, the ACT states that anxiety impairs cognitive performance by increasing the bottom-up, stimulus-driven, processing of threatening information. This manifests itself as increased attention to negative thoughts (worry) or to external stimuli (attentional bias to threat or threat-interference). Consequently, insufficient resources remain for the task at hand. Neurobiological evidence supports these cognitive theories. The bottom-up system, carried out mainly by subcortical areas [20,21,22], and the top-down system, mediated by the (dorso-) lateral prefrontal cortex (DLPFC; [20,23]), interact reciprocally as described by the ACT. High levels of stress-induced catecholamines in the PFC disrupt the balance in this dual-component system by activating the limbic salience attentional network, demonstrated as elevated attention to negative information, and simultaneously decreasing the effectiveness of PFC-mediated top-down control [8,20,21]. Although cognitive interference theories of CPA go a long way in explaining subjective clinical and experimental observations, their emphasis on cognitive processes of distraction may lead to a relative neglect of the direct biological effects of stress and anxiety on executive function. Anxiety/stress may also directly disrupt (DL)PFC, a brain region importantly involved in working memory (WM) and cognitive executive function [24,25,26], and thus executive cognitive (e.g., academic) performance [11,20,23,27].

The fact that anxiety impairs cognitive performance is well-documented (e.g., [14,21,28]; for a review on trait anxiety, see [29]).There is also ample evidence showing that acute stress, induced by the Trier Social Stress Test (TSST) or the Cold Pressure Test, impairs executive performance (e.g., [30,31,32,33,34]). However, there are also studies, using the same stress-induction procedures, that did not find any effects (e.g. [35,36,37]). Anxiety is also related to increased attentional bias for negative external or internal stimuli, such as worry-related thoughts (e.g., [12,15]). In the present study, the term threat-interference refers to the process of a threat interfering with executive task performance, an inhibitory process that is suggested to be the same for external or internal (verbal) stimuli [15]. However, the majority of previous findings concern trait anxiety. Trait anxiety refers to stable individual differences in anxiety predisposition, whereas state anxiety is an episodic mood state [12,38]. The trait-state anxiety theory [38] (Spielberger, 2013) suggests that trait anxiety moderates the vulnerability of individuals to stress, meaning that individuals experience feelings of anxiety only in situations that activate their state anxiety, while trait anxiety is associated with the degree to which a situation is evaluated as stressful. Trait and state anxiety are suggested to have distinct and interactive effects on executive cognitive performance ([39]; for empirical evidence, see [40]).

Although the effects of stress on threat-interference and cognitive performance have been investigated repeatedly, they have rarely been tested in a single study. Coy et al. [41] found that acute stress impaired objectively measured cognitive performance by increasing self-reported interference by negative thoughts. Another study showed that spatial attentional bias to threatening pictures, as assessed with a visual-probe task, mediated the relationship between state anxiety and WM performance [42]; however, state anxiety was not manipulated in that study. Although the above mentioned theories exist for more than two decades, no study has yet investigated the negative effects of acute stress on (objectively measured) threat-interference and cognitive performance simultaneously. Moreover, although threat-interference is suggested to play a crucial role in the context of CPA, it is noteworthy that very few studies have reported on objectively assessed threat-interference or even automatic attention toward threat in direct relation to CPA [43,44,45,46]. In the current study, we investigate the effects of acute CPA-related stress, in relation to trait CPA, on objectively measured cognitive performance and objectively measured interference of negative information during WM performance.

There are several experimental methods to induce state anxiety. Procedures with a large social-evaluative component, such as the TSST [47], reliably produce moderate to large changes in subjective and physiological parameters [48,49,50,51]. In the TSST, participants undergo a mock job interview and perform an arithmetic task in front of a committee. The TSST procedure is reliable but also somewhat impractical as it requires three additional experimenters. Furthermore, the participants are fully aware that the job interview is fake which could threaten the validity of a measurement, especially a self-report measure, due to demand effects. This validity issue may also apply to the mental arithmetic part of the TSST as it is introduced in the same context right after the fake job interview. On the other hand, in the L-PAST, participants are led to believe that their performance is genuinely being evaluated in field that is relevant to them, and a crucial difference of the L-PAST is that participants are not required to imagine or simulate the context in which the evaluation takes place, therefore increasing the validity of the test. It was vital to develop a test procedure with a stressor that matches CPA-related outcome measures. Therefore, we developed the Leiden-Performance Anxiety Stress Test (L-PAST). The L-PAST incorporates both crucial factors for laboratory stress-induction, i.e. social evaluation and uncontrollability [49]. The procedure consists of a face-to-face verbally-administered mental arithmetic task where the participants receive repeated scripted negative feedback in relation to peer-performance. Importantly, the procedure is developed in such a way to keep anticipation anxiety high even after the task by informing the participants that they will have to repeat the arithmetic test after completing an additional cognitive test.

Anxiety, and consequently also CPA, is a phenomenon that is manifested as a reactivity in emotionality (awareness of the heightened activation of the automatic nervous system) and cognition (worry) [19,52,53]. Worry can be defined as an uncontrollable cognitive response prior, during, and/or after an evaluative situation that represents a concern over forthcoming negative outcomes, such as worry over failure or negative evaluation (e.g., [54]). The trait cognitive test anxiety scale (CTAS) [52] is a validated instrument to assess the cognitive component of anxiety (worry) in test situations. Although the important role of cognitive anxiety and worry in CPA seems established theoretically, emotional, visceral anxiety must not be dismissed from the study of CPA, if only because of above mentioned direct effects of stress on PFC executive cognition [21]. We used a visual analogue scale (cf., [8]) to assess both features of CPA (cognitive and affective anxiety)–the State Performance Anxiety Scale (SPAS; Angelidis et al., in preparation).

In summary, the main goal of the study was to investigate the effects of CPA-related stress on objectively assessed cognitive performance and threat-interference during performance. In order to investigate this phenomenon, we induced CPA-related stress. A computerized WM task,–the commonly used *n*-back task–with emotionally charged word distracters, was used to assess threat-related interference during WM performance. Words were selected as distracters because words and worry-related thoughts share the same modality and are expected to compete for the phonological loop resources of the WM system, which is theorized to play a role in performance anxiety [54]. First, we expected that participants in the stress group would show impaired cognitive performance, in terms of reaction time and accuracy. Additionally, we hypothesized that participants in the stress group would show increased interference from negative evaluation stimuli during performance. We also expected that CTAS scores would moderate the effects of CPA-related stress on cognitive performance and threat-interference: the effects of stress on cognitive performance and threat-interference would be stronger for individuals with higher CTAS scores. Finally, we expected that all the moderating trait-state effects would be specific to CTAS and not general trait anxiety, assessed with the trait version of Spielberger’s State-Trait Anxiety Inventory (STAI-t) [55]. The second goal was to validate the new stressor for use in CPA research. We expected that higher levels of stress would be observed in the stress group than in the control group, as assessed with both subjective and objective measures: state performance anxiety, heart-rate activity, and salivary cortisol levels. Additionally, we hypothesized that trait CTAS scores would moderate the effect of the stress-induction procedure on state performance anxiety; participants with higher CTAS scores would show a larger increase in state performance anxiety after the stressor. These hypotheses were tested in a sample of college students for whom CPA and the substantive theme of the L-PAST and threatening distracters are personally relevant and ecologically valid.

## Materials and methods

### Participants

Eighty-six Dutch-speaking female participants were recruited from Leiden University campus and were randomly allocated to the control (*n* = 41) or stress group (*n* = 45). Only females were tested for both practical reasons and because females have higher average levels of test anxiety [3,4,56]. Exclusion criteria were self-report: presence of current mood, anxiety, or attention disorder; frequent or recent use of psychoactive substance; history of neurological disorder; any current major medical condition; use of cardiac or antihistamine medication; injuries (including small injuries in the mouth); recent change in the use of hormonal contraception; pregnancy; or lactation. The study was approved by the Psychology Research Ethics Committee in Leiden University (#2686632883).

### Apparatus and materials

#### Heart rate activity

Heart rate activity was assessed continuously with BIOPAC hardware and ACQKNOWLEDGE software. Three disposable electrodes (Kendall^TM^, Conductive Adhesive Hydrogel Foam Electrodes) were attached (after cleaning the skin with alcohol), placed right above the sternum, at the bottom left side of the chest, and the bottom right side of the chest (used as a reference), according to Einthoven’s triangle. Electrocardiographic data were recorded in a seated position and participants were discouraged to move excessively. Heart rate analysis was performed with MATLAB software. Offline high pass and low pass filters of 1 Hz and 50 Hz, respectively, were applied. Data were inspected visually, and faulty R-peaks were removed by an independent experimenter blinded to the conditions. For each participant, HR was averaged separately for the 5-min resting state measurement and during the two computerized tasks.

#### Salivary cortisol levels

Saliva samples were taken to observe changes in the endocrine stress response to the manipulation. Samples were collected with Salivette collection devices (Sarstedt, Nümbrecht, Germany) at five time points -3, +12, +22. +48, +58 min in relation to the onset of the stressor. It was made sure that participants kept the cotton swab in their mouth for two minutes to ensure that it was completely saturated. Samples were stored at -20 C° in a freezer until shipment. Biomedical analyses of free cortisol concentration in saliva were performed (Dresden Lab Service, Dresden, Germany) using immunoassay with chemiluminescence-detection (IBL-International, Hamburg, Germany).

#### Questionnaires

**Trait cognitive test anxiety.** The Cognitive Test Anxiety Scale (CTAS) [52] was used to assess trait cognitive test anxiety as the measure for CPA. The revised version of this scale includes 27 items (e.g., “*I worry more about doing well on tests than I should*”) rated on a 4-point Likert scale. The Dutch version of the Cognitive Test Anxiety Scale was used in our experiment, which has excellent internal consistency (Cronbach’s *α* = .93) (Putman & Jans, unpublished manuscript). In the present study, internal consistency was also excellent (Cronbach’s *α* = .93)

**Trait anxiety.** The trait subscale of Spielberger’s State-Trait Anxiety Inventory (STAI-t) [55,57] consists of 20 items (e.g., “*I feel nervous and restless*”) rated on a 4-point Likert scale. The internal consistency of STAI-t was good in the present study (Cronbach’s *α* = .89).

**Trait attentional control.** The Attentional Control Scale (ACS) [58,59] consists of 20 items (e.g., “*After being interrupted or distracted*, *I can easily shift my attention back to what I was doing before*”) rated in a 4-point Likert scale. Internal consistency of the total score was good in this study (Cronbach’s *α* = .77).

**State anxiety and state attentional control.** State performance anxiety and state attentional control were assessed with the state performance anxiety scale (SPAS) and the state attentional control scale (SACS), developed in our lab (Angelidis et al., in preparation), similar to Putman et al. [8]. The SPAS consisted of 7 items for state performance anxiety; 4 items for emotionality (e.g., “*I have a feeling of panic*”) and 3 items for cognition (e.g., “*I feel like a failure*”). Six items were included to assess state attentional control (e.g., “*I have trouble concentrating*”). See S1 Text for these 13 item. An additional 8 filler items were included (e.g., “*I am thirsty*”)–all 21 items were presented in a semi-random order. Participants had to respond on a visual analogue scale by crossing 100 mm lines, anchored “not at all” and “completely” to the left and right end. The internal consistency for state anxiety was *a* = .76 and for state attentional control *a* = .77.

#### *N*-back task with emotional distracters

**Fig 1:**
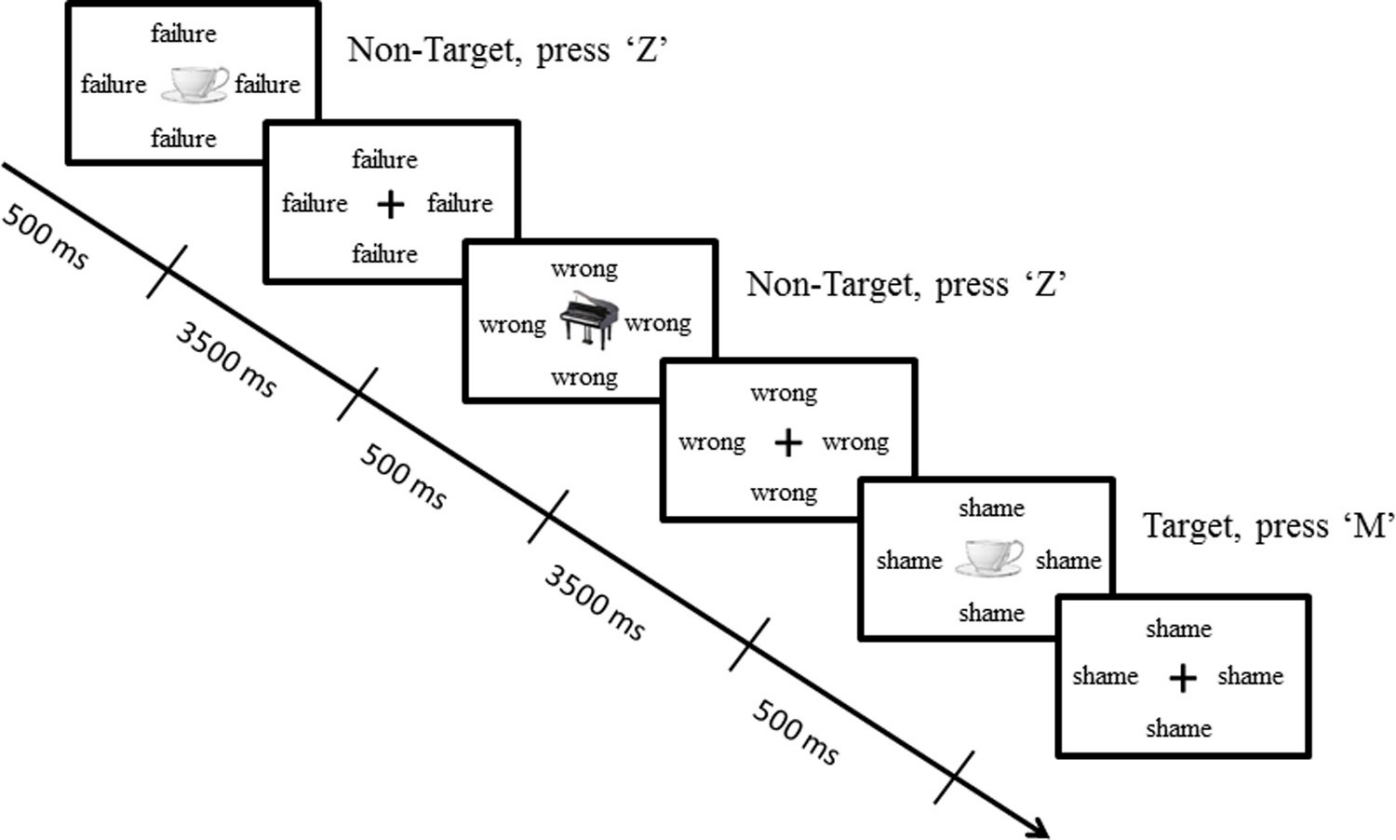
Illustration of the *n*-back task with emotional word distracters, depicting an example of a 2-back memory-load condition with negative word-distracters. Participants had to press the “M” key when the picture was the same as two pictures before while they had to press the “Z” key for the rest of the trials.

An *n*-back task with emotional distracters was developed, based on previous emotional n-back tasks (e.g., [60,61]), to assess working memory performance and interference by negative stimuli. Participants were asked to respond with a target button “M” or a non-target button “Z” to a pseudorandom presentation of simple pictures of objects with simultaneous random presentation of words as distracters. Two memory-load conditions were included in the present task: a 2-back memory load (e.g., press the target button whenever the current picture is identical to the picture presented two trials before) and a 3-back memory load (e.g., press the target button whenever the current picture is identical to the picture presented three trials before). See Fig 1 for an example of a 2-back working memory load. The task consisted of two rounds of six blocks each, that included an equal number of memory-load conditions and three emotional distracter-type conditions (no word, neutral words, and negative evaluation words).The blocks were pseudorandomly presented (i.e., each round began with a 2-back/no-distracter block to help participants adapt to the procedure of the task). Each block consisted of sixteen trials (five target trials). The first three trials were never target trials to ensure an equal number of trials for both memory-load conditions. We chose simple visual representations of common objects (e.g., chair, piano) rather than alphabetic characters that are more commonly used in *n*-back tasks, to prevent potential interference or facilitation of target alphabetic character processing by concurrent presentation of alphabetic characters in distracter words. These images of simple objects were selected from the 101 Caltech Object Categories [62] and presented in grey-scale and 140 × 140 pixels. Sixteen words per category were selected as distracters and were randomly presented within each block. Both word-categories were based on previous studies (subset of words used in, [44,63], and similar to these). See S1 Table for both categories of words. The neutral words were words such as “episode” or “frequency” while the negative words were related to exams and negative evaluation (e.g., “failure”, “incorrect”). Each word was presented surrounding the target pictures in bold, 28 pt. Courier New font. The two emotional categories of words were matched for length and frequency of use based on the Dutch database “INL 5 Miljoen Woorden Corpus” [64]. Each block started with a 500 ms blank screen, followed by the sixteen trials. Each trial started with a picture presentation in the center of the white background for 500 ms followed by a black fixation cross for 3500 ms. In the blocks with emotional distracters, a new distracter was displayed in the screen at the beginning of every trial and remained for the rest of the trial. The words of each category were randomly presented within the blocks. There was a break of one minute between the two rounds. During the task, participants were instructed to remain still and use their index fingers to press the two buttons. Participants were instructed to respond as quickly as possible and they were informed about the memory-load condition (2-back or 3-back) directly before each block. Detailed instructions about the task were provided during the practice session that consisted of four blocks of sixteen trials (five target trials). In the practice session, each memory-load condition was presented with no-word and neutral word distracter types. The neutral words of the practice block were different from the testing procedure. The task took approximately 25 min. Finally, the task was programmed in E-prime 2.0 and presented on a 22 inch monitor with a screen resolution of 1680 × 1050.

Performance was evaluated based on RTs of correct responses, and accuracy of target trials. Responses faster than 200 ms were excluded, resulting in the loss of 0.03% of the total trials. Additionally, responses above 3 s and ± 3 SDs from the average reaction time were removed from the emotional conditions (neutral and negative evaluation distracters), as in other studies assessing threat-interference (e.g., [65,66]), resulting in the loss of 1.3% of the total trials.

### Procedure

**Fig 2:**
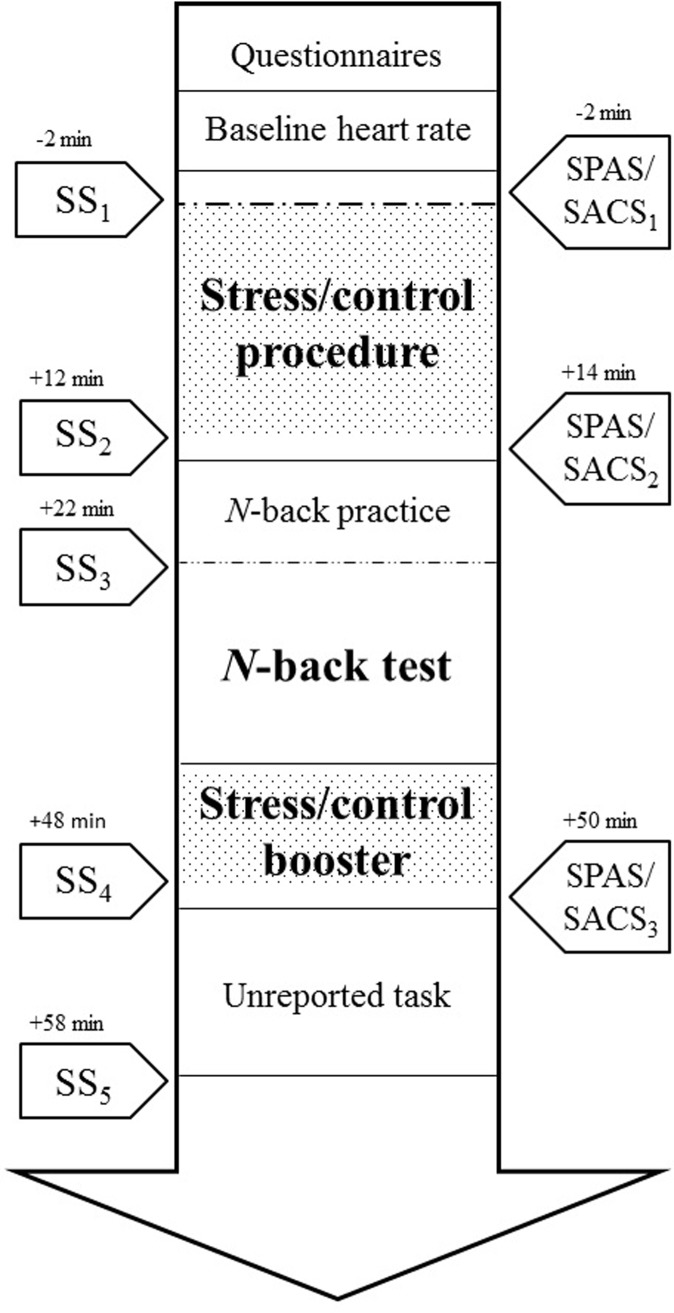
Illustration of the main components of the procedure. SS = saliva sample, SPAS = state performance anxiety, SACS = state attentional control. Time of SS and SPAS is reported in minutes in relation to the onset of the stress/control procedure.

All participants were tested individually between 1:15 p.m. and 5:15 p.m. to account for the diurnal variations of cortisol release [48]. Fig 2 illustrates the main components of the procedure. Participants first filled out the informed consent form, the consumption form and the trait questionnaires, which was followed by the 5-min habituation video and the baseline ECG measurement. After random assignment to the control or stress manipulation, participants performed the stress/control procedure and then the *n*-back task with emotional distracters. Next, participants went through a stress/control boost procedure (see S2 Text: a brief stress/control manipulation) followed by another cognitive task that is not relevant for the present research question and thus, it will not be further reported. State performance anxiety and state attentional control were assessed at three time points: right before the stress/control manipulation, before the test procedure of the *n*-back task, and before the second task. Saliva samples were taken before (-3 min with regard to the onset of the manipulation) and after the manipulation (+12 min), before the test procedure of the *n*-back task (+22 min), after the stress/control boost procedure (+48 min), and in the end of the procedure (+58 min). Participants were instructed not to drink anything but water and abstain from eating food, smoking, or brushing their teeth, and to minimize physical exercise during the hour prior to the experiment. They were also instructed not to drink more than two alcohol units on the evening prior to their participation. Written informed consent was provided by all participants. Information regarding the stress manipulation was initially withheld, but all participants were extensively debriefed at the end of the procedure (approved by the Psychology Research Ethics Committee in Leiden University).

#### Stress/control procedures

We used the Leiden Performance Anxiety Stress Procedure (L-PAST) to induce stress. This stressor is an adaptation of the one used in Putman et al. [8]. Participants had to perform a mental verbal arithmetic task under time pressure with scripted negative feedback in front of an experimenter. The scripted and bogus negative feedback was the same regardless of actual performance (the mental arithmetic task was in fact too difficult for the participants to be able to infer deception). Components of social evaluation were established by the presence of an experimenter and video-recording. Moreover, participants were told that their performance and other aspects of their behavior would be further evaluated by students. The mental arithmetic task and the subsequent computerized cognitive tasks were introduced under the same context of social evaluation. In order to ensure anticipation anxiety, participants were told that they would repeat the mental arithmetic test after completing another test. Moreover, in order to keep state anxiety levels high, a brief version of the aforementioned procedure (stress booster) was used after the *n*-back task. The control manipulation was similar to the stress manipulation (participants solved the same arithmetic questions) but without the elements of social evaluation and time-pressure. The stress/control procedures lasted approximately 10 minutes. See Supplementary Materials (S2 Text) for a detailed description of the stress and control procedures.

### Statistical analysis

T-tests were conducted to investigate potential group differences on background characteristics. A series of ANOVAs were performed to test the various hypotheses. First, we performed two 2 (Group: Control vs. Stress) × 3 (Time: t_1_ vs. t_2_ vs. t_3_) mixed ANOVAs for both self-report measures, state performance anxiety, and state attentional control. We then performed a 2 (Group: Control vs Stress) × 3 (Time: t_1_ vs t_2_ vs t_3_) mixed ANOVA for heart rate activity and a 2 (Condition: Control vs Stress) × 5 (Time: t_1_ vs t_2_ vs t_3_ vs t_4_ vs t_5_) mixed ANOVA for cortisol levels. In order to test whether CTAS moderated the effect of the stressor on state anxiety, a 2 (Group: Control vs Stress) × 3 (Time: t_1_ vs t_2_ vs t_3_) mixed ANCOVA was performed with CTAS as a covariate and state performance anxiety as an outcome measure.

In order to test the effect of the stressor on WM performance, we performed two 2 (Group: Control vs Stress) × 2 (Load: 2-back vs 3-back) mixed ANOVAs separately for reaction time (RT) and accuracy scores, for the no distracter condition. A 2-factor Bonferroni correction was used as we tested the same hypothesis separately for RT and accuracy. To test the moderating role of CTAS on the effect of the stressor on WM performance, the same mixed ANOVAs with CTAS as a covariate were performed separately for RT and accuracy, for the no distracter condition. A 4-factor Bonferroni correction was used to account for conducting the same analysis separately for RT and accuracy, and CTAS and STAI-t.

To test the effect of the stressor on threat-interference, two 2 (Group: Control vs Stress) × 2 (Load: 2-back vs 3-back) × 2 (Valence: Neutral vs Negative distracters) mixed ANOVAs were performed separately for RT and accuracy scores. A 2-factor Bonferroni correction was used as we tested the same hypothesis separately for RT and accuracy. To test the moderation effect of CTAS on threat-interference, the same mixed ANOVAs were performed with CTAS as a covariate separately for RT and accuracy. The direction of the moderation effects was further tested with post-hoc correlations. A 4-factor Bonferroni correction was used as we perform the same analysis separately for RT and accuracy, and CTAS and STAI-t. All significant interactions are further explored with post-hoc t-tests or correlations.

The power analysis showed that with a sample of *N* = 86, an *a* at .05, and a power of .80, we could detect small effect sizes (*f* = .12) for the analyses related to the main effects of stress, and medium effect size (*f* = .31) for the moderation analyses (G Power 3.1.6) [67].

## Results

No group differences were observed on background characteristics, trait characteristics, or baseline measurements of state anxiety, heart rate activity, and salivary cortisol levels (see Table 1). Sixty-three percent of the control group used hormonal contraception methods (46% used oral contraceptives and 17% used birth control implants, intrauterine devices, or vaginal rings) while the percentage was 74% for the stress group (67% used oral contraceptives and 7% used birth control implants, intrauterine devices, or vaginal rings). A positive correlation was observed between CTAS and STAI-t, *r* = .46, *p* < .001. A negative correlation was observed between STAI-t and ACS, *r* = -.52, *p* < .001, as commonly reported (e.g., [9,68,69]). A negative correlation was observed between CTAS and ACS, *r* = -.45, *p* < .001.

**Table 1:** Means (and standard deviations) and t-tests of background characteristics, and self-report and objective measurements of stress for the control (*n* = 41) and the stress group (*n* = 45).

	Control	Stress	*p*	*d*
Age	21.0 (2)	21.6 (2.2)	.249	0.25
Education	6.3 (1.8)	6.3 (1.8)	.946	0.02
Contraception	63%	74%	.248	0.001
CTAS	57.7 (15.5)	60.0 (13.2)	.471	0.16
STAI-t	35.9 (7.1)	37.6 (8.4)	.322	0.22
SPA_1_	20.8 (11.2)	21 (12.6)	.927	0.02
SPA_2_	23.4 (12.4)	39.9 (19)	< .001	1.02
SPA_3_	18.8 (12.1)	44.4 (22.4)	< .001	1.41
AC_1_	58.1 (12)	57.1 (12.6)	.721	0.08
AC_2_	56.9 (11.6)	44.3 (15.5)	< .001	0.92
AC_3_	60.6 (12.5)	40.7 (17.3)	< .001	1.11
HR_1_	77.6 (10.7)	79.1 (11.5)	.533	0.14
HR_2_	76.5 (9.5)	81.1 (12.5)	.032*	0.41
HR_3_	76.1 (9.3)	80.2 (12.4)	.044*	0.37
Cortisol_1_	8.2 (3.6)	8.3 (3.8)	.961	0.01
Cortisol_2_	7.7 (4)	8 (3.9)	.728	0.08
Cortisol_3_	7.1 (3.5)	8.7 (4.6)	.033	0.40
Cortisol_4_	5.7 (2.6)	7.5 (3.5)	.006	0.61
Cortisol_5_	5.4 (2.4)	7.4 (4.2)	.005	0.62

Reported descriptives of cortisol levels are not Ln-normalized for more intuitive appreciation and comparability with other studies. Education = a score of 6.3 reflects a university level in the Dutch academic system, Contraception: use of hormonal contraception methods, CTAS = trait cognitive test anxiety, STAI-t = Spielberger's state trait anxiety inventory—trait subscale, SA = state anxiety, AC = attentional control,

SA_1_/AC_1_ = before manipulation,

SA_2_/AC_2_ = before the test-procedure of the *n*-back task,

SA_3_/AC_3_ = after the booster,

HR_1_ = baseline heart rate activity in bpm,

HR_2_ = HR during the *n*-back task,

HR_3_ = HR activity during the second task after the booster,

Cortisol_1_ = baseline salivary cortisol levels in nmol/l (3 min prior to the onset of the manipulation),

Cortisol_2_ = cortisol levels 12 min after the onset of the manipulation,

Cortisol_3_ = right before the test procedure of the *n*-back task (+22 min),

Cortisol_4_ = after the booster (+48 min),

Cortisol_5_ = at the end of the procedure (+58 min),

*one-tailed, *p* < .05.

Regarding the most relevant correlations for the state VAS measurements, a negative correlation was observed between baseline state anxiety and state attentional control, *r* = -.53, *p* < .001. Moreover, a positive association was observed between baseline state attentional control and ACS, *r* = .50, *p* < .001.

### Stress-manipulation check

Means and post-hoc *t*-tests are presented in Table 1.

#### Self-report measures

**State performance anxiety.** Analysis revealed a significant Group × Time interaction, *F*(2, 83) = 25.732, *p* < .001, η_*p*_^2^ = .383. State anxiety scores did not differ at t_1_ while the stress group showed increased levels of state anxiety after the manipulation, at both t_2_ and t_3_ (see Table 1).

**State attentional control.** Analysis revealed a significant Group × Time interaction, *F*(2, 83) = 10.561, *p* < .001, η_*p*_^2^ = .296. State attentional control scores did not differ at t_1_, while the stress group showed decreased levels of state attentional control after the manipulation, at both t_2_ and t_3_ (see Table 1). Thus, the manipulation was successful in decreasing the level of state attentional control in the stress group.

#### Objective measures

**Heart rate.** Due to technical problems, heart rate data of two participants in the stress group could not be analyzed and were excluded from the relevant analyses. Since the assumption of sphericity was violated, *χ*^*2*^ = 37.95, *p* < .001, the degrees of freedom were corrected using Greenhouse-Geisser’s estimates of sphericity (*ε* = .728). Analyses revealed a significant effect of Group × Time interaction, *F*(1.456, 119.353) = 4.276, *p* = .027, η_*p*_^2^ = .05. Heart rate did not differ at resting-state between the two groups, whereas it was higher in the stress group after the manipulation, at both t_2_ and t_3_ (see Table 1).

**Cortisol levels.** Analyses, controlling for use of hormonal contraception methods, were performed on ln-normalized data. Since the assumption of sphericity was violated, *χ*^*2*^ = 244.22, *p* < .001, the degrees of freedom were corrected using Greenhouse-Geisser’s estimates of sphericity (*ε* = .395). Analyses revealed a significant Group × Time interaction, *F*(1.580, 131.141) = 12.274, *p* < .001, η_*p*_^2^ = .129. The significant interaction indicates that the two groups did not differ at t_1_ and t_2_, while the stress group had higher cortisol levels than the control group at t_3_, t_4_ and t_5_ (see Table 1).

Thus, the stressor was effective in increasing cortisol levels, heart rate, and self-report state anxiety as compared to the control group.

### Moderation analyses for the role of trait CPA on state performance anxiety

Analyses revealed a significant Time × Group × CTAS interaction, *F*(2, 81) = 10.513, *p* < .001, η_*p*_^2^ = .206. The Time × Group × CTAS interaction remained significant after controlling for STAI-t, *F*(2, 80) = 10.609, *p* < .001, η_*p*_^2^ = .210. The same analysis was performed only with STAI-t as a covariate revealing a non-significant Time × Group × STAI-t interaction, *F*(2, 81) = 0.185, *p* = .831, η_*p*_^2^ = .005. Thus, results confirm that CTAS has a unique moderating effect on the stressor-state anxiety relationship.

In order to test whether CTAS moderated the effect of the stressor on SA at t_2_ and/or t_3_ compared to t_1_, separate mixed 2 × 2 rm ANOVAs were conducted for t_2_ and t_3_. The crucial Time (t_1_ vs t_2_ and t_1_ vs t_3_ respectively) × Group × CTAS interaction was significant for both t_2_ and t_3_, *F*(1, 83) = 18.207, *p* < .001, η_*p*_^2^ = .182, and *F*(1, 83) = 19.629, *p* < .001, η_*p*_^2^ = .193, respectively.

**Fig 3:**
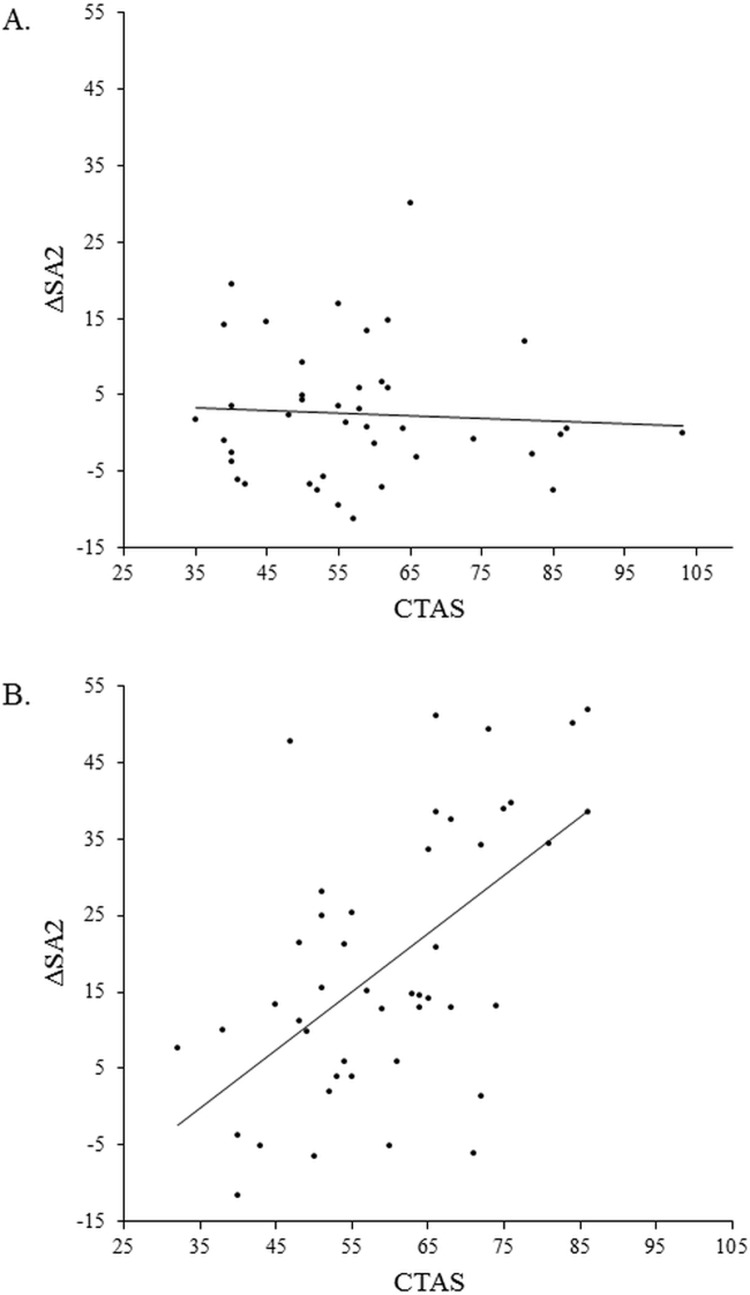
Scatterplots for the relationships between CTAS and ΔSA_2_ (SA at t_2_ minus t_1_) in the control (panel a; *r* = -.06, *p* = .711) and the stress group (panel b; *r* = .57, *p* < .001).

We ran separate correlations between CTAS and the contrast scores, ΔSA_2_ (state anxiety at t_2_ minus state anxiety at t_1_) and ΔSA_3_ (state anxiety at t_3_ minus state anxiety at t_1_), for the two different groups in order to unravel the nature of the interactions. Larger values indicate larger increases in state anxiety after the manipulation. CTAS correlated positively with both ΔSA_2_ and ΔSA_3_ in the stress group, *r* = .57, *p* < .001 and *r* = .66, *p* < .001 respectively, whereas the correlations were not significant in the control group, *r* = -.06, *p* = .711 and *r* = .29, *p* = .068. See Fig 3 for scatterplots of these relations. The results confirm that the effect of group on state performance anxiety was moderated by CTAS: participants with higher CTAS scores, compared to participants with lower CTAS scores, showed a higher increase of state performance anxiety in the stress group.

### Effect of the stressor on WM performance

**RT.** Analysis revealed a main effect of Load, *F*(1, 84) = 21.872, *p* < .001, η_*p*_^2^ = .207, indicating that participants were slower in the 3-back load (*M* = 876, *SD* = 237) compared to the 2-back load (*M* = 759, *SD* = 275), and a main effect of Group, *F*(1, 84) = 5.954, *p* = .034, η_*p*_^2^ = .066 (a 2-factor Bonferroni correction was applied), indicating that the stress group (*M* = 880, *SD* = 302) was slower during the WM task compared to the control group (*M* = 740, *SD* = 192).

**Accuracy.** Analysis showed a main effect of Load, *F*(1, 84) = 55.458, *p* < .001, η_*p*_^2^ = .398, indicating that participants were less accurate in the 3-back load (*M* = 72%, *SD* = 18%) compared to the 2-back load (*M* = 87%, *SD* = 15%). No other main or interaction effects were significant. Thus, stress did not affect response accuracy.

Thus, the stress group was slower on WM performance as compared to the control group. See Table 2 for detailed scores on *n*-back performance.

**Table 2:** Means (and standard deviations) of *n*-back performance for the control (*n* = 41) and the stress groups (*n* = 45).

	Load	Distracter type	Control	Stress
RT				
	2-back			
		No distracter	690 (207)	821 (315)
		Neutral	802 (257)	841 (262)
		Negative	748 (228)	818 (271)
	3-back			
		No distracter	803 (210)	942 (348)
		Neutral	885 (278)	1005 (350)
		Negative	875 (295)	1030 (361)
Accuracy (%)				
	2-back			
		No distracter	87 (15)	87 (16)
		Neutral	83 (16)	81 (17)
		Negative	79 (18)	77 (21)
	3-back			
		No distracter	73 (20)	71 (17)
		Neutral	71 (22)	71 (22)
		Negative	72 (21)	67 (19)

### Moderation analyses for the role of trait CPA on WM performance

**Fig 4:**
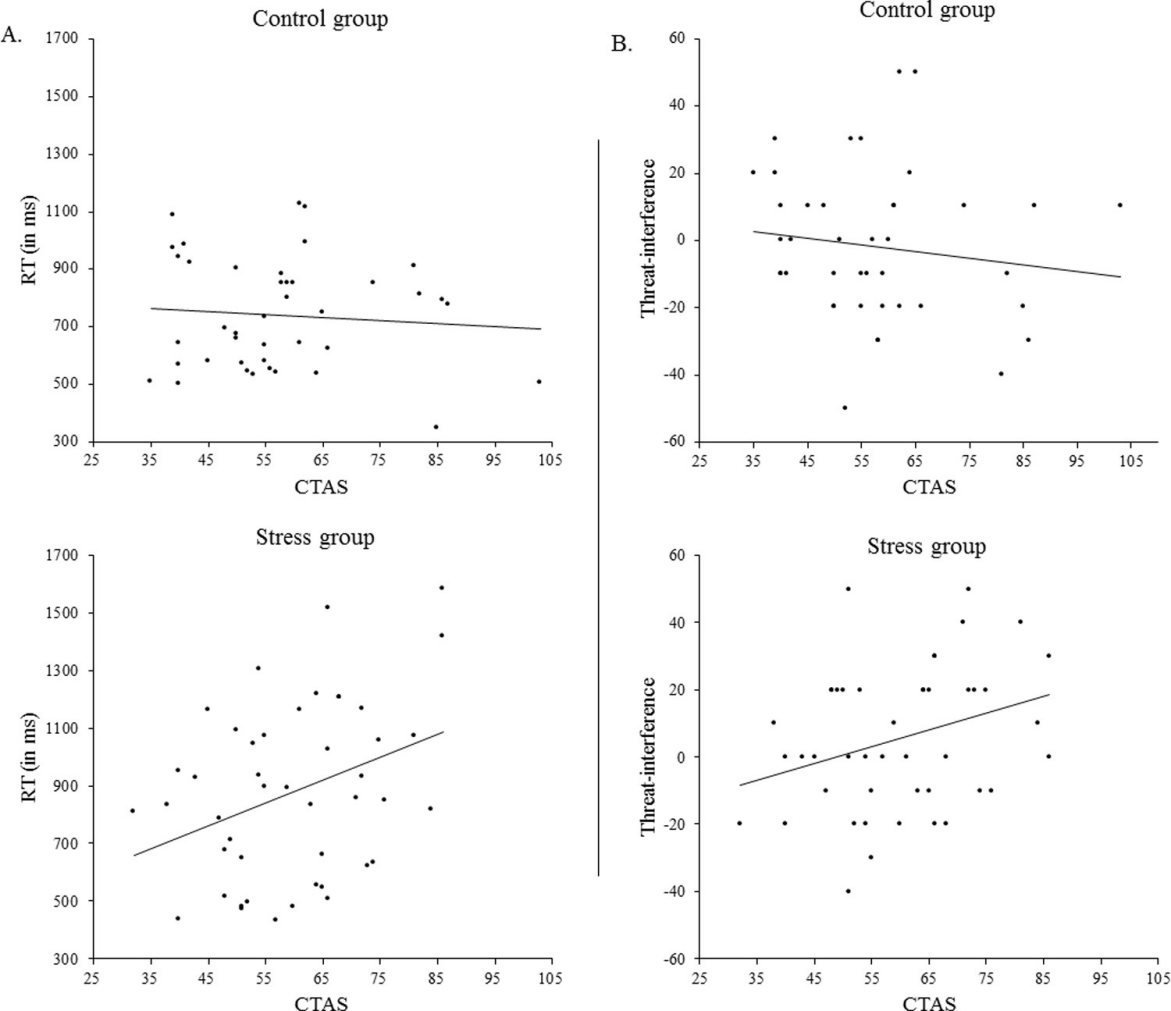
Scatterplots for the relationship between CTAS and *n*-back performance in the control and stress groups. A) Scatterplots for the relationship between CTAS and RT during the blank condition in the control (upper panel; *r* = -.08, *p* = .618) and the stress group (lower panel; *r* = .35, *p* = .02). B) Scatterplots for the relationship between CTAS and threat-interference during the high load condition as assessed by accuracy (accuracy scores during neutral condition. minus accuracy scores during negative evaluation condition in %) in the control (upper panel; *r* = -.138, *p* = .388) and the stress group (lower panel; *r* = .312, *p* = .037).

**RT.** Analyses revealed a significant Group × CTAS interaction, *F*(1, 82) = 6.726, *p* = .044 (corrected for a 4-factor Bonferroni correction), η_*p*_^2^ = .076. The correlations between CTAS and overall RT in *n*-back task are *r* = .35, *p* = .02 for the stress group and *r* = -.08, *p* = .618 for the control group (see Fig 4A). Thus, the effect of stress on WM performance, as assessed with RT, was stronger for participants with higher CTAS scores compared to participants with lower CTAS scores. The Group × CTAS interaction remained significant, *F*(1, 81) = 7.034, *p* = .010, η_*p*_^2^ = .080, after controlling for STAI-t. The same analysis was performed with STAI-t as a covariate that did not yield any significant effects (all relevant *p* values > .309). Thus, the effect of stress on overall RT was unique for CTAS.

**Accuracy.** No significant main effects or interactions were found. Thus, CTAS did not moderate the effect of stress on response accuracy. The results were similar when controlling for STAI-t.

Thus, CTAS scores moderated the effect of stress on WM performance as assessed with reaction times: in the stress group, participants with higher CTAS scores were slower on WM performance compared to participants with lower CTAS scores.

### Effect of the stressor on threat-interference

**RT.** Analyses revealed a main effect of Load, *F*(1, 84) = 65.561, *p* < .001, η_*p*_^2^ = .438, reflecting overall slower RT during the 3-back load (*M* = 927, *SE* = 30) compared to the 2-back load (*M* = 788, *SE* = 24). No other interactions or main effects were significant.

**Accuracy.** Analyses revealed a main effect of Load, *F*(1, 84) = 37.400, *p* < .001, η_*p*_^2^ = .308, indicating that participants were more accurate in the 2-back load (*M* = 80%, *SD* = 15%) compared to the 3-back load (*M* = 70%, *SD* = 17.5%). No other interactions or main effects were significant.

Thus, the stress group alone did not affect threat-interference on WM performance.

### Moderation analyses for the role of trait CPA on threat-interference

**RT.** Analyses revealed a significant Group × CTAS interaction, *F*(1, 82) = 7.265, *p* = .036, η_*p*_^2^ = .081 (a 4-factor Bonferroni correction was applied). The correlations between CTAS and average RT for both distracter types, in order to clarify the Group × CTAS interaction, are *r* = .32, *p* = .033 for the stress group and *r* = -.24, *p* = .134 for the control group. Thus, CTAS moderated the effect of stress on RT during both distracter conditions, as in the no-distracter condition; participants in the stress group with higher CTAS scores than participants with lower CTAS scores, were slower during WM performance with distracters, regardless of their valence. The Group × Valence × CTAS interaction, *F*(1, 82) = 0.146, *p* = .704, η_*p*_^2^ = .002, was not significant, and thus, our hypothesis that stress would affect threat-interference, as assessed with RTs, was not confirmed.

**Accuracy.** Analyses revealed a significant Group × Valence × Load × CTAS interaction, *F*(1, 82) = 6.678, *p* = .048, η_*p*_^2^ = .075 (a 4-factor Bonferroni correction was applied). In order to disentangle the 4-way interaction, we ran separate mixed ANCOVAs for the two memory load conditions. While no significant results were observed for the 2-back memory-load condition, in the 3-back condition, analysis revealed a significant Group × Valence × CTAS interaction, *F*(1, 82) = 4.599, *p* = .035, η_*p*_^2^ = .053.

The association between CTAS and threat-interference (ACC during neutral condition minus ACC during negative condition), with higher scores indicating more errors (in %) during test with negative evaluation words as distracters compared to neutral words, was *r* = .312, *p* = .037 for the stress group. This indicated that individuals with higher CTAS scores in the stress group made more errors during negative distraction words compared to the neutral distraction words (see Fig 4B). The same association was negative and not significant for the control group (*r* = -.138, *p* = .388). The Group × Valence × Load × CTAS interaction remained significant after controlling for STAI-t, *F*(1, 81) = 5.709, *p* = .019, η_*p*_^2^ = .066. The same analysis was repeated only with STAI-t revealing a non-significant relevant interaction, Group × Valence × Load × CTAS: *F*(1, 82) = 0.118, *p* = .732, η_*p*_^2^ = .001.

Overall, CTAS moderated the effect of stress on interference from negative evaluation words during WM performance, as assessed by accuracy: in the stress group, participants with higher CTAS scores, compared to participants with low CTAS, made more mistakes during the presence of negative evaluation words. This effect was uniquely explained by CTAS and not STAI-t.

## Discussion

The main goal of this study was to investigate the effects of acute cognitive performance anxiety (CPA) on objectively assessed cognitive performance and threat-interference. As expected, the stress group was slower during the WM task, and trait cognitive test anxiety (CTAS) scores moderated this effect. Specifically, individuals with higher CTAS scores were slower under stress. Moreover, CTAS scores moderated the effect of stress on threat-interference during a WM task as assessed by accuracy in the 3-back memory load condition: individuals with higher CTAS scores performed worse during trials with negative evaluation distracters as compared to trials with neutral distracters. Our method to induce and assess performance-like stress produced the intended effect: the stress group indeed had higher subjective and objective stress scores after the stress manipulation than the control group. Moreover, CTAS moderated this effect; specifically, the stressor was more effective in individuals with higher CTAS scores. Trait anxiety did not moderate these effects.

The main purpose of this study was to investigate the effects of CPA-related stress on objectively assessed threat-related interference during cognitive performance and cognitive performance. We found that stress impaired self-reported attentional control (cf., [8]) and increased RT during WM performance with and without (emotional) distraction. Stress also increased interference from negative evaluation-related stimuli during high load WM performance in individuals with higher trait cognitive test anxiety. These findings are in line with the attentional control theory which posits that anxiety affects cognitive performance by disrupting attentional control, as manifested in interference from negative stimuli [14,19]. The role of threat-interference on stress-induced cognitive impairments is commonly assumed and supported by studies using self-report measures (e.g., [41]), making our study the first to simultaneously demonstrate the negative effects of a controlled acute stress induction procedure (i.e., the L-PAST) on objectively assessed cognitive performance and threat-interference. This objective assessment of threat-interference is crucial. Although introspection of cognitive processes is used in various studies, it has limited validity, specifically given the issue of the aforementioned demand characteristics. Even though the L-PAST is likely less subject to this threat to validity because of its obtrusive nature, the objective assessment of threat-interference is especially important as the Cognitive Interference Theory [19] is so face-valid, even for lay people. Thus, evidence based on objective assessment is likely firmer than previous studies using only self-report.

We also found that trait CPA moderated the effects of stress on cognitive performance, as assessed by RT, and threat-interference during high WM load, as assessed by accuracy. Specifically, stress resulted in slower responses and higher threat-interference during WM performance for participants with higher trait cognitive test anxiety. These results are in line with previous studies suggesting the importance of both high trait and state anxiety in threat-interference [39,40,70] and performance [71,72]. However, previous evidence were not always in relation to acute stress. This is the first study showing that trait CPA moderated both effects in relation to acute stress. These effects were uniquely explained by trait cognitive test anxiety and not general trait anxiety, as assessed by STAI-t. These results, together with the moderating effect of trait CPA on state anxiety, further support the validity of our stress induction procedure (L-PAST). The fact that the individuals in the stress group, who reported to be more worried in performance-evaluative settings, showed higher levels of stress, slower performance during the WM task, and higher interference from negative evaluation-related words strongly suggests that L-PAST, indeed, induces CPA-related stress.

Furthermore, stress affected cognitive performance as assessed by reaction times but not when assessed by accuracy. This is in line with cognitive theories [14,73] supporting the notion that anxiety exacerbates the amount (quantity) of resources required to reach a certain level of performance rather than the quality of the performance, as indicated by accuracy. The longer reaction times are explained by the lack of available resources for the task at hand, due to increased worrisome thoughts, induced by the L-PAST. Similarly, there is empirical evidence of the effects of acute stress on *n*-back performance as assessed by reaction times but not accuracy [33,74]. Other studies, however, found effects of stress on reaction time- and accuracy-based *n*-back performance (e.g., [75]). Moreover, another study [76], similarly to our study, did not find an effect of stress, as induced by aversive sounds, on accuracy-based *n*-back performance, suggesting that the effects of stress on effectiveness were compensated by enhanced motivation and cognitive resources. The literature thus provides inconclusive empirical evidence. In the present study, trait cognitive test anxiety had a moderating effect on stress-induced threat-interference in terms of accuracy but only in a higher load condition (3-back condition). This load-specificity is in line with the load theory and its supporting empirical evidence [77,78,79] suggesting that higher cognitive load diminishes the resources of cognitive control over goal-relevant information, resulting in increased interference from salient distracters. Stress challenges capacity of cognitive control [20,21,23], and it is therefore expected to result in increased interference from salient stimuli when cognitive load is high. Empirical evidence has previously shown that stress affects WM performance during higher cognitive loads but not when the load is low [80]. Moreover, pharmacological studies have shown that manipulation of stress-related hormones, resulting in enhanced cognitive control, lead to reduced threat-interference during WM performance, only when cognitive demands are high [81,82]. The lack of threat-interference as assessed by reaction times might be due to the high variability of responses, considering the difficulty of the task. Nevertheless, the aforementioned cognitive theories (i.e. attentional control theory and cognitive interference theory) were developed mainly on the basis of results related to simple cognitive tasks in regards to threat-interference, in which cognitive load was not manipulated. Threat-interference is traditionally assessed by reaction time tasks such as the emotional Stroop task, in which participants need to name the color of presented words (e.g., [39,65,66]), and the dot-probe task (e.g., [83,84,85]), which is suggested to assess spatial attentional bias to threatening stimuli by indicating the position of a probe following the simultaneous presentation of a neutral and a threatening stimulus. Both tasks have been used in the context of CPA (e.g., [43,44,45,46]). However, these tasks may be less valid for our research questions as they may be too easy to assess the influence on more complex higher order executive cognition, as might be required for a more ecologically valid approach to the phenomenon of CPA. Besides, the dot-probe paradigm does not assess interference during performance but rather spatial attention. Finally, it was observed that significant effects of WM load on RTs and accuracy were non-significant in a model controlling for CTAS and its interactions. This could perhaps suggest that WM load effects are also to some extent dependent on individual differences in trait cognitive test anxiety. However, since there was no significant interaction between CTAS and WM load, this remains a speculation.

In the present study, we developed an *n*-back task with emotional word distracters with the goal of increasing the ecological validity of the measurement in relation to CPA. The main advantage of this task is the possibility to manipulate the cognitive load. This could be important considering that the manipulation of cognitive load limits the available cognitive resources for the task at hand, resulting in threat-interference, in accordance with the load theory [78,79]. As expected, we found stress-induced threat-interference only in the condition with higher cognitive load. As WM memory capacity differs across age, especially the ability to control task-irrelevant information (e.g., [86,87,88]) the *n*-back task is a useful tool in investigating threat-interference during performance when testing individuals of different ages. This is particularly of importance to test anxiety research, considering that such anxiety clearly develops early in a child’s academic career but often remains a problem throughout adulthood as well [3,89]. Moreover, in the *n-*back task, we used emotional word-distracters as they share the same modality with worry-related thoughts, and thus they both compete for resources of the phonological loop of WM capacity. According to Eysenck et al. [54], objectively assessed threat-interference could best be addressed by challenging the same slave system of WM [90], rather than, for instance, using visual stimuli as distracters for the quasi-verbal processing of worrying thoughts [91]. By using word-distracters, we also managed to use distracters that are thematically relevant to CPA, such as words related to academic performance and negative evaluation. In general, we believe that the L-PAST, in combination with the emotional *n*-back task, is a valuable method to investigate the effects of stress on cognition, especially in the context of acute CPA.

As expected, the L-PAST increased stress levels compared to the control group, when assessed by subjective and objective measurements. This is in line with a review suggesting that public speaking or verbal interaction tasks that include elements of social evaluation and loss of control are crucial in inducing stress [49]. The cortisol and heart rate reactivity were limited compared to other studies. This may be explained by the inclusion of participants using a hormonal contraception method or the hormonal cycle, both which are known to affect cortisol response to stress. However, the majority of the current sample used hormonal contraception methods that are known to suppress cortisol reactivity. Thus, it could be expected that L-PAST would be more effective in a sample of naturally cycling females. Results showed that cortisol levels dropped in the control group from t_1_ to t_5_ (likely due to the diurnal HPA cycle and physiological inactivity; c.f. [92,93,94], but cortisol levels in the stress group decreased significantly less and were significantly higher than in the control group at t_3_-t_5_ This indicates that the psychosocial L-PAST stressor resulted in greater HPA activity in the stress group than in control group, verifying that the L-PAST was effective. Interestingly, in the stress group cortisol even increased from t_1_ to t_3_ but was lower again at t_4_. Some studies using the TSST stressor seem to report longer lasting HPA increases (e.g., [35,93]). A possible explanation may be that the L-PAST procedure lasts shorter than the TSST. However, one should not over-interpret cortisol changes within the stress group; the crucial outcome is the comparison between groups. Regarding heart rate activity, even though the effect of L-PAST was not as previously reported for TSST, it should be mentioned that any effect on heart rate was controlled for postural effect. In many TSST studies where participants need to stand during the stress procedure, the participants are seated in the control condition and thus, the reported effects on heart rate may be confounded by posture, unlike in our method. It is also worth mentioning that heart rate activity is analyzed during performance of our cognitive tasks and not during the stress procedure indicating that participants were stressed during cognitive performance and not just during the stress procedure. A key element of the L-PAST is the induction of anticipatory anxiety by “warning” participants of impending extra rounds of the scripted arithmetic test after the cognitive tests. This was also a critical factor in maintaining high stress levels for a longer period of time, up to 60 min after the onset of the stressor. The limited time-window to assess performance under stress is a common practical limitation in studies of acute stress. In this manner, the L-PAST has the added advantage of assessing the effects on performance of multiple or long-lasting tasks. Another advantage of the L-PAST is that it is easily administered and it can be executed by a single main experimenter. The main purpose of developing the L-PAST was to create a more ecologically valid state for the evaluation of intellectual performance, such as CPA-related stress. The results support this by revealing that the effects of the stressor on state performance anxiety were moderated by trait CPA, an effect that remained after the stress booster (45 min after the onset of the stressor). Specifically, participants with a predisposition to become cognitively anxious in test-related conditions indeed showed higher levels of stress after our stress manipulation. Importantly, this moderation effect, as well as the moderating effects on WM performance and threat-interference, was uniquely explained by the trait CPA and not trait anxiety (STAI-t) in general. These findings are in line with the trait-state anxiety theory and empirical evidence suggesting that individuals with high levels of trait anxiety will experience more stress in a personally threatening situation [38,95]. The present sample scored on average within a moderate range of CTAS according to Thomas, Cassady, and Finch [96] and the CTAS scores were close to the average (*M* = 60, *SD* = 13.3, *median* = 59) of a large Dutch-speaking student sample (*n* = 998; unpublished data from our lab). Similarly, the STAI-t scores of our sample were similar to other student samples (e.g., [8,66,84]) and thus, the unique effects of CTAS, relative to STAI-t, could not be explained by the samples’ vulnerability in one of the two measurements. All in all, the L-PAST is an easily-administered, ecologically-valid standardized lab stressorfor CPA, such as test anxiety (the standardized protocol of the L-PAST can be obtained from the authors).

Although the present sample of health college-aged females is relevant for the research of (C)PA and the nature of the L-PAST, as university students are constantly evaluated for their cognitive performance, the main limitation of this study is its external validity. Even though the present sample was at moderate range of CTAS [96], it was still consisted by females who are suggested to be more anxious [3,4,56]. As a result, our sample might be more susceptible to the stress procedure and the subsequent *n*-back task. Nonetheless, it was observed that within the present sample, females with higher CTAS scores are more susceptible to the stress procesure and *n*-back performance. Furthermore, the phenomenon of CPA occurs at every age and also in clinical populations and thus, the present finding should be investigated in males but also in clinical samples and people of different age range. Moreover, in the current study, participants were included irrespective of use of contraception method or hormonal cycle which are known to affect cortisol levels in response to stress-induction procedures. In addition, the effects of CPA-related stress on WM performance and threat-interference were stronger for participants with higher CTAS scores. Future studies should investigate the generalizability of the present findings in individuals preselected for high scores on CTAS or a measure of related construct, such as social anxiety, of which (C)PA is considered a qualifier [97,98], but also in different populations (e.g., school-aged students and clinical populations). Furthermore, it would be of interest to test the effect of stress on threat-interference during WM performance with higher cognitive load, as the effect was present only for the higher load condition (3-back) and for people with high CTAS scores. This would further shed light on the role of cognitive load in relation to threat-interference. Moreover, we used an *n*-back task, measuring WM performance and interference thereon from failure-related words. Use of such objective performance measures can, of course, not prove directly that stress-effects on performance are indeed mediated by interference from those words or distracting thoughts that these may trigger, although this class of methods is generally assumed to measure such effects (e.g., [15,99]). Ultimately, progress in this field of study probably requires combinations of objective and self-report measures such as thought-probing (e.g., [100,101]) or subjective report of task-interfering thoughts (e.g., [102]). Finally, further research should also focus on the biological mechanisms of CPA-related stress and cognition. The effects of stress on cognition are partially explained by the glucocorticoid and the noradrenergic system (e.g., [11,21]). Thus, it would be interesting to investigate whether pharmacological interventions, targeting these systems, could prevent the negative effects of CPA on cognition.

In summary, it is concluded that acute CPA-related stress increases interference from negative evaluation during performance while impairing attentional control and WM performance. Moreover, the present evidence suggests that we have developed a useful experimental method to induce and assess stress-induced cognitive deficits in the context of CPA. Finally, these results underline the importance of negative cognition, worry over performance or negative evaluation, on the stress-performance relation, and they further highlight the necessity of its objective assessment on the field of (C)PA.

## Supporting information

S1 Text Supplementary materials for items (in Dutch and English) used in state performance anxiety scale (SPAS) and state attentional control scale (SACS). The number next to every item represents the presented order.
(DOCX)

S1 Table Supplementary materials for detailed report of the two categories of words, in Dutch and their translation in English, used as distracters in the *n*-back task. (DOCX)

S2 Text Supplementary materials for detailed description of stress /control procedures. (DOCX)
