# Supplementary material for: I’m going to fail! Acute cognitive performance anxiety increases threat-interference and impairs WM performance
Source: PLoS One. 2019 Feb 7;14(2):e0210824. doi: 10.1371/journal.pone.0210824 (PMC6366876; doi:10.1371/journal.pone.0210824)
Supplement: S1 Text — The number next to every item represents the presented order. (DOCX) [file pone.0210824.s001.docx]

**S1 File.**

**Supplementary materials for items (in Dutch and English) used in State Performance Anxiety Scale (SPAS) and State Attentional Control Scale (SACS). The number next to every item represent the presented order.**

SPAS:

- Items for cognitive component of anxiety

I feel like a failure. (13)

Ik voel me een mislukking.

I am worried about how I am performing. (19)

Ik maak me zorgen over hoe ik presteer.

I am concerned I won’t be able to concentrate. (21)

Ik maak me zorgen dat ik me niet kan concentreren.

- Items for emotional component of anxiety

I am stressed and my heart is racing. (6)

Ik ben gestrest en mijn hartslag is verhoogd.

I feel a sense of panic. (14)

Ik heb een gevoel van paniek.

I feel agitated. (15)

Ik voel me geagiteerd.

I’m nervous and my palms are sweaty. (18)

Ik ben nerveus en mijn handpalmen zweten.

SACS:

I can easily shift my attention between different things. (4)

Ik kan gemakkelijk mijn aandacht verschuiven tussen verschillende dingen

I have difficulty concentrating. (7; reversed score)

Ik heb moeite me te concentreren.

I feel very focused. (9)

Ik voel me erg gefocust.

I feel distracted. (11; reversed score)

Ik voel me afgeleid.

It is easy for me to block out distracting thoughts. (16)

Het is gemakkelijk voor mij om afleidende gedachten te blokkeren.

I feel capable of processing new information. (20)

Ik voel me bekwaam om nieuwe informatie te verwerken.
