# Supplementary material for: I’m going to fail! Acute cognitive performance anxiety increases threat-interference and impairs WM performance
Source: PLoS One. 2019 Feb 7;14(2):e0210824. doi: 10.1371/journal.pone.0210824 (PMC6366876; doi:10.1371/journal.pone.0210824)
Supplement: S1 Table — (DOCX) [file pone.0210824.s002.docx]

**S2 File.**

**Supplementary materials for detailed report of the two categories of words, in Dutch and their translation in English, used as distracters in the *n*-back task.**

| Neutral |  | Negative evaluation |  |
| --- | --- | --- | --- |
| Dutch | English | Dutch | English |
| proporties | proportions | mislukking | failure |
| ritme | rhythm | falen | to fail |
| nut | use/purpose | dom | stupid |
| afdruk | print | gezakt | failed |
| vlakte | flatland/plain | afgaan | to loose face |
| afkorting | abbreviation | schaamte | shame |
| episode | episode | idioot | idiot/idiotic |
| meten | to measure | fout | wrong/error |
| frequentie | frequency | beoordeeld | evaluated/judged |
| samenhangen | to correlate/cohere | zenuwachtig | nervous |
| afbeelden | picture/to depict | vernederd | humiliated |
| formaat | size | nerveus | nervous |
| aanpak | approach | kritiek | criticism |
| omvang | magnitude | angst | fear/anxiety |
| omvangrijk | extensive | frustratie | frustration |
| rubriek | written column | onjuist | incorrect |
