# Supplementary material for: I’m going to fail! Acute cognitive performance anxiety increases threat-interference and impairs WM performance
Source: PLoS One. 2019 Feb 7;14(2):e0210824. doi: 10.1371/journal.pone.0210824 (PMC6366876; doi:10.1371/journal.pone.0210824)
Supplement: S2 Text — (DOCX) [file pone.0210824.s003.docx]

**S3 File.**

**Supplementary materials for detailed description of stress /control procedures**

The Leiden Performance Anxiety Stress Procedure (L-PAST) was used to induce stress. Participants went through a mental verbal arithmetic task with blocks of increasing difficulty. Each block consisted of five multiple choice-questions, presented by the experimenter and the participant had 6 s to respond. False expectations were given to the participants by telling them that most students reach at least the 4^th^ level. However, most participants were expected to struggle by the 2^nd^ level as it was designed to be sufficiently difficult without realizing that they were getting scripted negative feedback). After the participants’ “failure” to proceed to the next level, they were offered a second chance, which then ended with the same negative result. The experimenter had a distant and strict attitude. In order to induce anticipatory stress, participants were told that they would continue with another test related to intelligence (to frame the following cognitive tasks as more relevant to their concerns) and that they would repeat this arithmetic task directly after the other cognitive test. The whole procedure, including the cognitive tasks after the stressor, was video recorded and right before the beginning of the bogus arithmetic task, participants had to introduce themselves to the camera by telling their name, average grade in their studies, and additional personal information. Moreover, before the beginning of the bogus arithmetic task, participants received instructions, similar to Coy et al. (2011), that are known to induce stress. The instructions introduced the bogus arithmetic task but also the subsequent cognitive tasks under a context of evaluation: they were informed that they would perform several intelligence test that are related to academic performance and their future career, and the camera recording would be used by other students to evaluate their cognitive performance “on other dimensions” and compare it with other students. Right before every task, participants received a brief stressful instruction such as “Do your best, we need to get a good impression of your performance level” or “Do your best. Afterwards, you will perform the arithmetic task one more time”. The stress procedure lasted approximately 10 minutes. After the first two computerized tasks, the second part of L-PAST was applied to make sure that the stress levels stay high. During the brief stress booster, participants performed the second level again, including new test questions, and they again received false negative feedback. Finally, they were told that they would have another chance to do better after the next cognitive test in order to sustain anticipatory anxiety.

The control manipulation was similar to the stress manipulation. Participants had to solve the same arithmetic questions but any evaluative elements were excluded. They were told that the purpose of the tasks was not to rate their performance but to validate the arithmetic questions because “we did not know what level of performance participants like them could reach”. The task was computerized and participants performed without the presence of the experimenter or a camera. Also, the participants did not have the same time pressure as in the stress procedure, and they received 15 s to answer the multiple choice questions. If the time was insufficient, they could respond with an additional response option, namely that the sum was too difficult. Before the beginning of the task, similar instructions as in Coy et al. (2011) were provided to ensure that participants did not feel that they were under evaluation but they should still aim for their optimal performance. Before every task, participants received brief instructions to boost their performance such as “Stay relaxed and try to do your best.” The duration was the same as the stress procedure. The same characteristics were applied for the control booster procedure.
